# Distinct autoregulatory roles of ELFN1 intracellular and extracellular domains on membrane trafficking, synaptic localization, and dimerization

**DOI:** 10.1016/j.jbc.2024.108073

**Published:** 2024-12-13

**Authors:** Henry A. Dunn, Simran K. Dhaliwal, Chu-Ting Chang, Kirill A. Martemyanov

**Affiliations:** 1 Department of Neuroscience, The Herbert Wertheim UF Scripps Institute for Biomedical Innovation & Technology, University of Florida, Jupiter, Florida, USA; 2 Department of Pharmacology and Therapeutics, University of Manitoba, Winnipeg, Canada; 3 Division of Neurodegenerative Disorders, St Boniface Hospital Albrechtsen Research Centre, Winnipeg, Manitoba, Canada; 4 The Skaggs Graduate School of Chemical and Biological Sciences, The Scripps Research Institute, Jupiter, Florida, USA

**Keywords:** membrane trafficking, synapse, dimerization, cell adhesion, C-terminal domain (carboxyl tail domain, CTD), N-terminal domain (amino terminal domain), trans-synaptic adhesion molecule

## Abstract

Synaptic adhesion molecules are essential components of the synapse, yet the diversity of these molecules and their associated functions remain to be fully characterized. Extracellular leucine rich repeat and fibronectin type III domain containing 1 (ELFN1) is a postsynaptic adhesion molecule in the brain that has been increasingly implicated in human neurological disease. ELFN1 is best known for trans-synaptically modulating group III metabotropic glutamate receptors (mGluRs). However, little is known about ELFN1 organization and regulation, which likely govern and precede its ultimate trans-synaptic engagement with group III mGluRs. Herein, we report that the intracellular ELFN1 domain controls membrane trafficking and post-synaptic localization of ELFN1. We pinpoint a ∼30 amino acid juxtamembranous region required for membrane-targeting and discover that ELFN1 exists as an obligate homodimer prior to its trafficking to the membrane. We determine that ELFN1 homodimerization is not appreciably affected by the intracellular region and instead utilizes the extracellular leucine rich repeats (LRR) domain. We find that a single membrane-targeting motif located in one protomer is sufficient for effective trafficking of the ELFN1 homodimer. We further demonstrate that the closest ELFN1 homolog, synaptic adhesion molecule ELFN2, exhibits similar properties and participates in heterodimerization with ELFN1. This establishes distinct autoregulatory roles of ELFN1 intracellular and extracellular domains on membrane trafficking, post-synaptic localization, and dimerization while indicating conservation of the mechanisms across the ELFN subfamily of cell adhesion molecules.

Synaptic adhesion molecules are critical for all aspects of synaptic function, including the establishment of synaptic contacts, recruitment and assembly of synaptic machineries, functional specification of synaptic properties, and the operational plasticity of the synapse (1, 2). In alignment with these essential roles, disruption of synaptic adhesion molecules has been highly implicated in a diverse array of neurological, neurodevelopmental, neuropsychiatric, and neurodegenerative diseases (3, 4, 5). Consequently, extensive focus is being placed on determining how synaptic adhesion molecules are dynamically regulated to orchestrate synaptic function at the inter- and intramolecular level.

Extracellular leucine rich repeat and fibronectin type III domain containing 1 (ELFN1) is a postsynaptic adhesion molecule in the brain known for regulating presynaptic release probabilities *via* trans-synaptic interactions with group III metabotropic glutamate receptors (mGluRs) (6, 7, 8, 9, 10, 11, 12). In the retina, ELFN1 similarly recruits group III mGluR trans-synaptically at photoreceptor synapses to enable transmission of the light signal (13, 14). A close homolog of ELFN1 with a distinct expression profile, ELFN2, has been identified and shown to play a similar role in controlling group III mGluRs in the retina and the brain (8, 14). Together, ELFN1 and ELFN2 constitute a two-member subfamily of ELFN cell adhesion molecules involved in controlling glutamate signaling in the nervous system.

In recent years, ELFN1 has been increasingly implicated in human neurological disease etiology, including epilepsy (12, 15, 16), autism (12, 15), post-traumatic stress disorder (17, 18), attention-deficit/hyperactivity disorder (12), intellectual disability (16), and schizophrenia (19). Interestingly, human pathogenic and disease-correlational ELFN1 mutations have been identified throughout the ELFN1 protein, including intracellular regions that could not directly contribute to ELFN1’s known function involving trans-synaptic interactions with group III mGluRs (12, 15, 16). Therefore, it has become necessary to investigate the unexplored function(s) of the intracellular domain, with the hypothesis that ELFN1 may host important autoregulatory functions that play an essential role in its localization and function.

Our knowledge of ELFN1 function has been exclusively tied to its ability to trans-synaptically modulate group III mGluRs *via* the long ELFN1 extracellular domain, yet the functional role of the extensive intracellular domain (∼400 amino acids) is unknown. In this study we test a hypothesis that the ELFN1 intracellular tail may provide an essential autoregulatory function. Indeed, we report that the ELFN1 intracellular domain plays a role in membrane trafficking and ultimate postsynaptic localization of ELFN1. In the process, we identify a ∼30 amino acid juxtamembranous region within ELFN1 essential for these trafficking processes that shares homology with a homodimerization domain of another protein. Therefore, we subsequently examine the ability of ELFN1 to homodimerize and investigate whether this homodimerization occurs before or after ELFN1 membrane trafficking. We further extend our observations to ELFN2 to show that it relies on similar principles. Collectively, we are able to present novel autoregulatory functions of ELFN proteins prior to their ability to engage group III mGluRs trans-synaptically.

## Results

### ELFN1 intracellular domain is essential for membrane trafficking and post-synaptic localization

**Figure 1:**
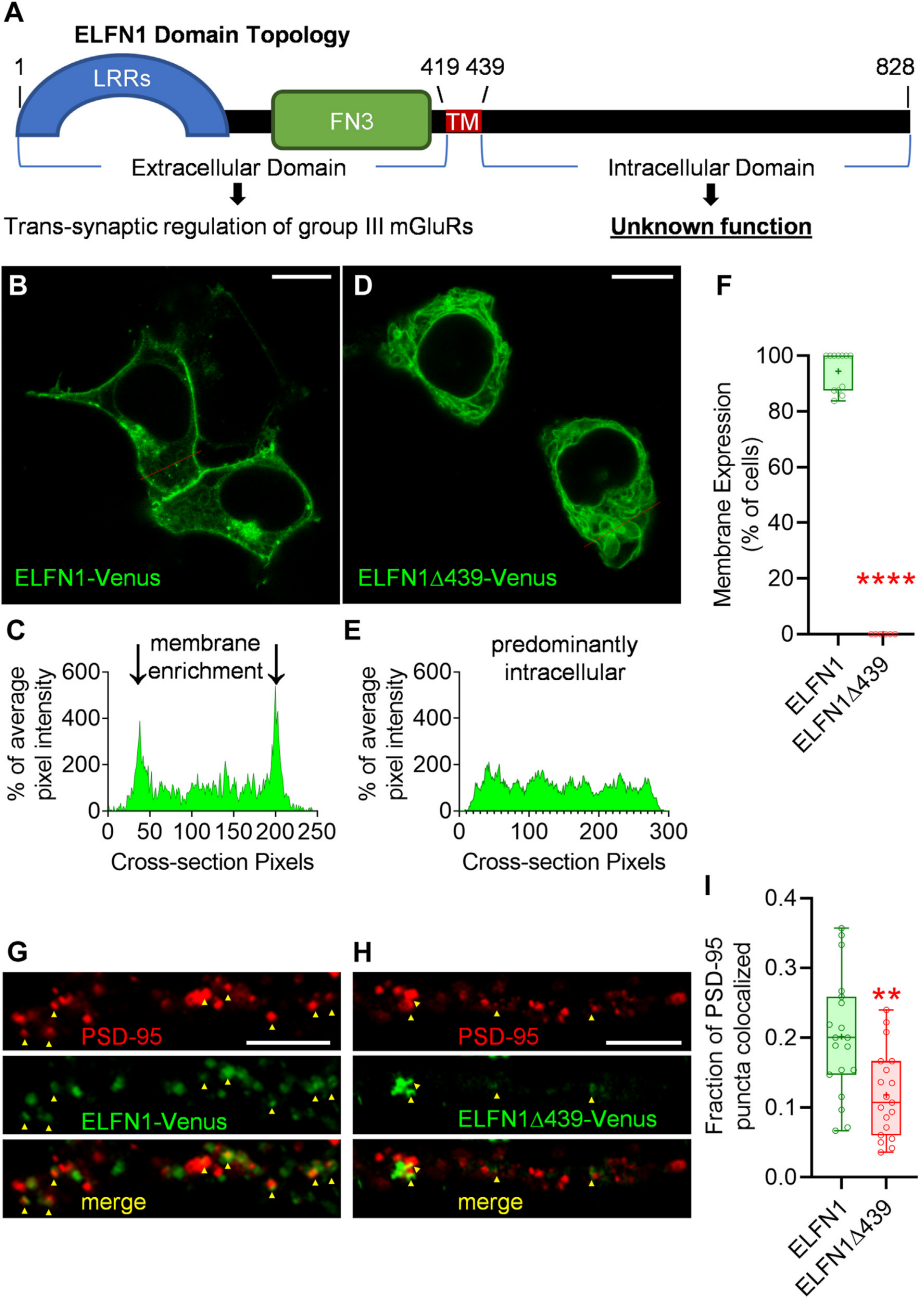
**ELFN1 intracellular domain is essential for membrane trafficking and post-synaptic localization.***A,* Schematic representation of ELFN1 extracellular and intracellular domains. *B,* ELFN1-Venus expressed in live HEK293 cells visualized using fluorescent confocal microscopy (scale = 10 μm). Red line indicates cross-section analyzed *(C)* showing the percentage of average pixel intensity is pronounced at the outer membrane region. *D,* ELFN1Δ439-Venus expressed in live HEK293 cells visualized using fluorescent confocal microscopy (scale = 10 μm). Red line indicates cross-section analyzed in *(E)* showing the percentage of average pixel intensity is consistent throughout the cell body, indicative of intracellular expression. *F,* Image quantification of percentage of cells exhibiting membrane enrichment. ELFN1-Venus (mean 94.45 ± SEM 2.011, n = 12, referring to images from independent transfections) was significantly different than ELFN1Δ439-Venus (mean 0.000 ± SEM 0.000, n = 6) using a two-tailed, unpaired Mann-Whitney test (∗∗∗∗*p* value < 0.0001) due to failed normality test. *G,* ELFN1-Venus or *(H)* ELFN1Δ439-Venus transfected in mouse primary cortical neurons, fixed and permeabilized for immunolabelling endogenous PSD-95 (scale = 10 μm). *I,* Quantification of the fraction of PSD-95 puncta colocalized with ELFN1-Venus (mean 0.2014 ± SEM 0.0198, n = 19) or ELFN1Δ439-Venus (mean 0.1182 ± SEM 0.0142, n = 19) demonstrated significant difference using a two-tailed, unpaired *t* test (∗∗*p* value = 0.0016) due to passed normality test.

The transmembrane region of ELFN1 is flanked by the N-terminal extracellular domain, which has an extensively characterized role in regulating group III mGluRs, and the C-terminal intracellular tail with an unknown role (Fig. 1*A*). To investigate the role of the ELFN1 intracellular domain, we engineered Venus fluorescent protein-tagged cDNA constructs of ELFN1 with intact or completely truncated carboxyl-termini: ELFN1-Venus and ELFN1Δ439-Venus, respectively. Using fluorescent confocal microscopy in human embryonic kidney 293 (HEK293) cells, ELFN1-Venus was found enriched at the membrane (Fig. 1*B*): as exemplified by the average pixel intensity measuring highest at the edge of the cell (Fig. 1*C*). Strikingly, ELFN1Δ439-Venus, which lacks the entire carboxyl terminus, was expressed predominantly intracellularly with no membrane enrichment (Fig. 1, *D* and *E*). Across transfected populations, ELFN1-Venus was found enriched at the membrane in >90% of cells, whereas ELFN1Δ439-Venus was never found to be enriched at the membrane (Fig. 1*F*).

To determine the relevance of these observations to the native neuronal environment, we studied post-synaptic targeting of ELFN1 in cultured primary mouse cortical neurons. Following ELFN1-Venus or ELFN1Δ439-Venus transfection, their localization was determined in reference to endogenous PSD-95 which demarks post-synaptic densities (Fig. 1, *G* and *H*). Quantification of ELFN1 co-localization with PSD-95-positive puncta revealed ∼50% reduction in the presence of ELFN1Δ439-Venus at the synapse when compared to “wild-type” ELFN1-Venus (Fig. 1*I*). These observations indicate that the intracellular domain possesses determinants essential for ELFN1 trafficking to the membrane and post-synaptic density.

### ELFN1 intracellular juxtamembrane region is required for plasma membrane trafficking

**Figure 2:**
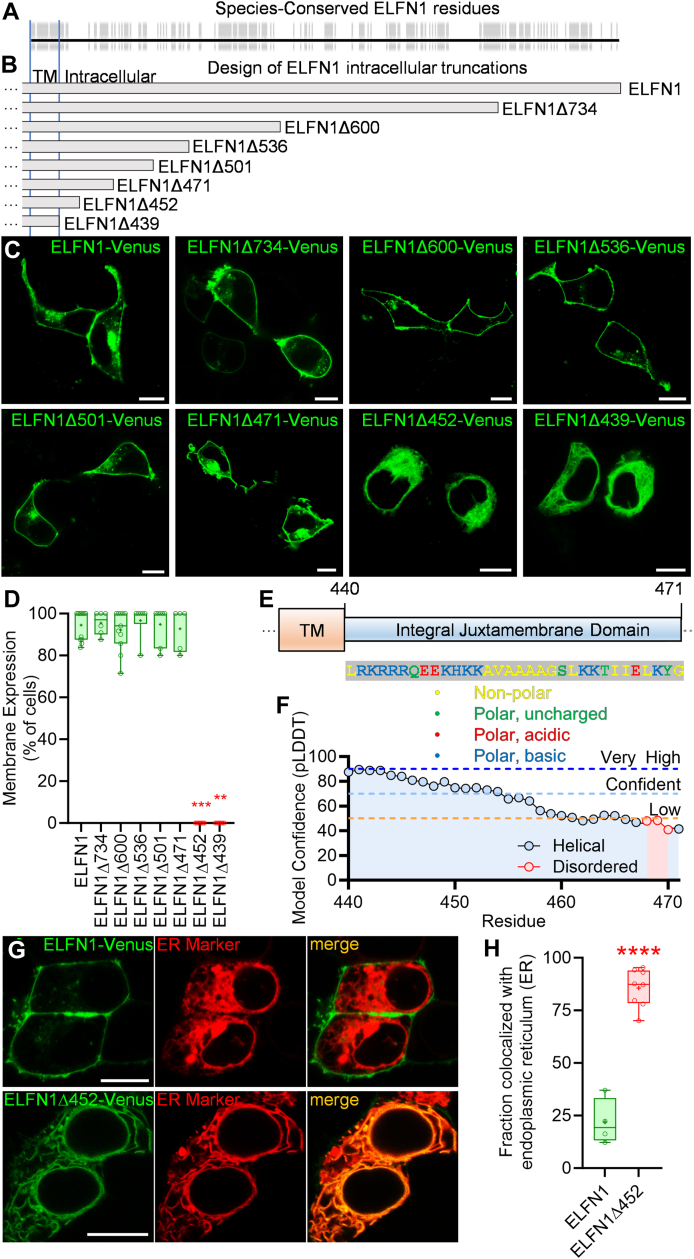
**ELFN1 intracellular juxtamembrane region is required for plasma membrane trafficking.***A,* Multiple sequence alignment of ELFN1 transmembrane (TM) and intracellular domain across 26 mammalian species, with conserved residues highlighted in grey. *B,* Structure-based design of ELFN1 constructs with various truncations of the intracellular tail ultimately culminating in a Venus tag. *C,* ELFN1-Venus full-length and truncated constructs expressed in live HEK293 cells visualized using fluorescent confocal microscopy (scale = 10 μm). *D,* Image quantification of percentage of cells exhibiting membrane enrichment. ELFN1-Venus (mean 94.45 ± SEM 2.011, n = 12 representing images from independent transfections) was not significantly different than ELFN1Δ734-Venus (mean 95.42 ± SEM 2.219, n = 6), ELFN1Δ600-Venus (mean 92.08 ± SEM 2.916, n = 11), ELFN1Δ536-Venus (mean 96.67 ± SEM 3.333, n = 6), ELFN1Δ501-Venus (mean 94.76 ± SEM 3.401, n = 7), or ELFN1Δ471-Venus (mean 92.67 ± SEM 4.522, n = 5) using a Kriskal-Wallis test and Dunn’s multiple comparisons test (*p* value > 0.9999) due to failed normality tests, but was significantly different compared to ELFN1Δ452-Venus (mean 0.000 ± SEM 0.000, n = 7) and ELFN1Δ439-Venus (mean 0.000 ± SEM 0.000, n = 6) using the same tests (∗∗∗*p* value = 0.0008, ∗∗*p* value = 0.0017, respectively). *E,* Schematic depiction of juxtamembranous sequence required for membrane localization with description of amino acids. *F,* Graphical depiction of AlphaFold ELFN1 structural model with residues within predicted helical structure depicted in blue, and disordered residues depicted in red. Model/per-residue confidence score – predicted Local Distance Difference Test (pLDDT) – is graphed on the y-axis. *G,* ELFN1-Venus full-length or ELFN1Δ452-Venus co-expressed with endoplasmic reticulum marker mCherry-SEC61 B in live HEK293 cells visualized using fluorescent confocal microscopy (scale = 10 μm). *H,* Quantification of the fraction of ELFN1-Venus pixels colocalized with mCherry-SEC61 B (mean 21.95% ± SEM 5.428%, n = 4 representing cells from independent experiments) or ELFN1Δ452- Venus pixels colocalized with mCherry-SEC61 B (mean 85.66% ± SEM 3.178%, n = 8) demonstrating significant difference using a two-tailed, unpaired *t* test (∗∗∗∗*p* value = <0.0001) due to passed normality test.

To determine the exact sequence required for ELFN1 trafficking, we performed a multiple sequence alignment of ELFN1 across 26 mammalian species (Fig. 2*A*) and used conserved regions as a guide for ELFN1 construct design (Fig. 2*B*). A panel of intracellular truncations of ELFN1-Venus varying in length were then evaluated for their localization in HEK293 cells by live-cell confocal fluorescent microscopy. Surprisingly, deletion of a large intracellular portion of ELFN1 had no effect on its localization, with ELFN1Δ734-Venus, ELFN1Δ600-Venus, ELFN1Δ536-Venus, ELFN1Δ501-Venus, and ELFN1Δ471-Venus all trafficking to the membrane in >90% of cells: akin to full-length ELFN1-Venus (Fig. 2, *C* and *D*). In contrast, ELFN1Δ452-Venus and ELFN1Δ439-Venus were not localized at the plasma membrane (Fig. 2, *C* and *D*). These results indicate that the plasma membrane-targeting determinant in ELFN1 is contained in the juxtamembrane sequence between amino acid residues 440 and 471. Analysis of this sequence revealed that it consists mostly of polar-basic and non-polar residues (Fig. 2*E*). AlphaFold structural modelling indicates that the proximal part of the region is mostly helical in nature, while the distal part is likely disordered (Fig. 2*F*). To determine the intracellular location of truncated ELFN1, ELFN1Δ452-Venus was co-expressed with endoplasmic reticulum marker mCherry- SEC61 B and was found to exhibit ∼85% colocalization: significantly higher than ELFN1-Venus colocalization with mCherry-SEC61 B of ∼20% colocalization (Fig. 2, *G* and *H*). These data suggest ELFN1’s intracellular juxtamembrane region is integral for membrane trafficking from the endoplasmic reticulum.

### ELFN1 exists as an obligate homodimer independent of its intracellular domain

**Figure 3:**
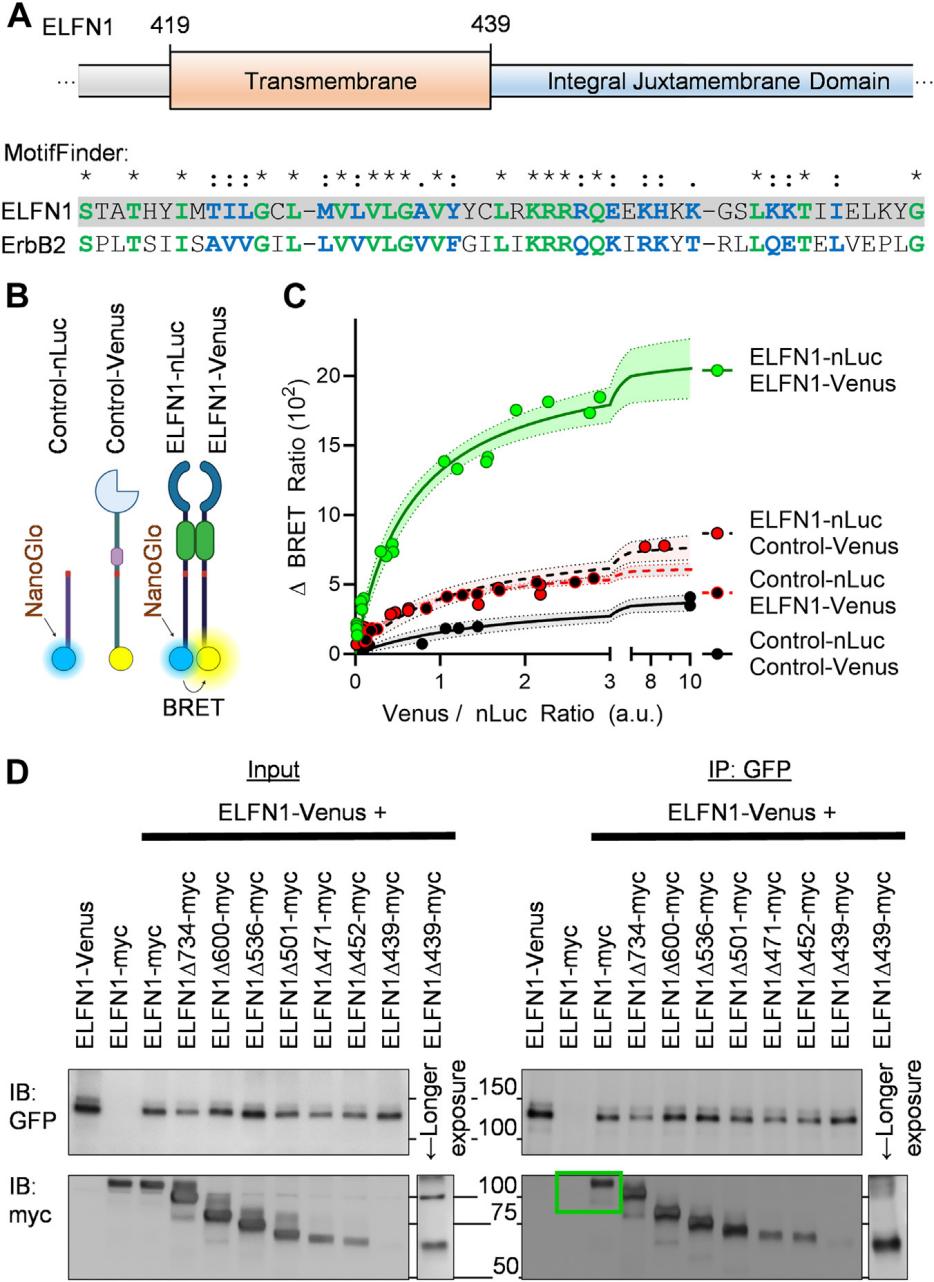
**ELFN1 exists as an obligate homodimer independent of its carboxyl terminal tail.***A,* Schematic depiction of MotifFinder analysis identifying homology of juxtamembranous and transmembrane ELFN1 region with ErbB2 homodimerization-domain: provoking the hypothesis for ELFN1 homodimerization. *B,* Schematic representation of nLuc- and Venus-tagged constructs used for *(C)* titration BRET experiments demonstrating increasing Control-Venus levels does not lead to an appreciable increase in BRET ratio with Control-nLuc (Bmax = 3.564–5.364). Neither control-nLuc with increasing ELFN1-Venus levels (Bmax = 6.042–6.980), nor ELFN1-nLuc with increasing Control-Venus levels (Bmax = 7.412–9.891) were significantly distinguishable from Control-nLuc with Control-Venus (adjusted *p*-value = 0.7275 and 0.6452, respectively). ELFN1-nLuc transfected with increasing amounts of ELFN1-Venus exhibited a change in BRET ratio (Bmax = 19.70–24.67) distinguishable from Control-nLuc with Control-Venus suggestive of ELFN1 homodimerization. Each non-linear regression includes 8 to 16 independent values across 4 independent experiments and error bars representing 95% confidence intervals. *D,* Representative Western blots from 4 independent co-immunoprecipitation experiments with lysate inputs expressing the described cDNAs on the left, and immunoprecipitations with protein G sepharose beads with GFP antibody on the right, indicating ELFN1-myc co-immunoprecipitated with ELFN1-Venus (green box). All myc-tag truncated ELFN1 constructs similarly co-immunoprecipitate with ELFN1-Venus, suggesting a lack of involvement of the intracellular domain in homodimerization. Longer exposure added for ELFN1Δ439-myc due to comparably lower expression levels.

To further evaluate properties of the integral juxtamembrane domain and its contribution to ELFN1 function, we utilized MotifFinder (https://www.genome.jp/tools/motif/) to search for similar sequences across the proteome (Fig. 3*A*). Interestingly, we found that this region bears similarity with a corresponding region from receptor tyrosine-protein kinases, particularly erythroblastic oncogene B2 (ErbB2) (Fig. 3*A*). Because ErbB2 as with other receptor tyrosine kinases are known to dimerize *via* this region (20), we hypothesized that ELFN1 may also exist as a dimer.

We first tested monomerization of ELFN1 using bioluminescence energy resonance transfer (BRET) strategy (Fig. 3*B*). We transiently transfected HEK293 cells with a constant level of nanoluciferase (nLuc)-tagged energy donor constructs (Control-nLuc or ELFN1-nLuc) and increasing levels of Venus-tagged acceptor constructs (Control-Venus or ELFN1-Venus). These donor/acceptor titration experiments showed a sharply increasing and saturating ΔBRET signal (*in green*) when ELFN1-nLuc was transfected with increasing amounts of ELFN1-Venus (Fig. 3*C*). In contrast, neither Control-nLuc transfected with increasing ELFN1-Venus levels, nor ELFN1-nLuc with increasing Control-Venus levels were distinguishable from Control-nLuc with Control-Venus: all of which exhibited significantly lower BRET ratios suggestive of non-specific interactions greater than 10 nm traditionally required for BRET (Fig. 3*C*). Although these live-cell BRET experiments support the hypothesis of ELFN1 homodimerization, this technique is not immune to false interpretation of dimerization state (21).

To further validate ELFN1 homodimerization, we conducted co-immunoprecipitation experiments where we analyzed the ability of ELFN1-Venus to pulldown ELFN1-myc following their co-expression in HEK293 cells. Indeed, we found that ELFN1-Venus was able to co-immunoprecipitate ELFN1-myc (Fig. 3*D*, *green box*). To determine the role of the intracellular domain in ELFN1 homodimerization, the same experiment was performed with a series of myc-tagged ELFN1 carboxyl terminal truncations (Fig. 3*D*). ELFN1Δ439-myc demonstrated lower expression than the other constructs, therefore longer exposures were used for both the lysate and co-immunoprecipitation blots. Interestingly, all truncated ELFN1-myc constructs co-immunoprecipitated with ELFN1-Venus (Fig. 3*D*). These experiments further suggest ELFN1 homodimerization and that it is not mediated by the intracellular domain of the protein.

### ELFN1 homodimerization utilizes the extracellular LRR domain and precedes membrane localization

**Figure 4:**
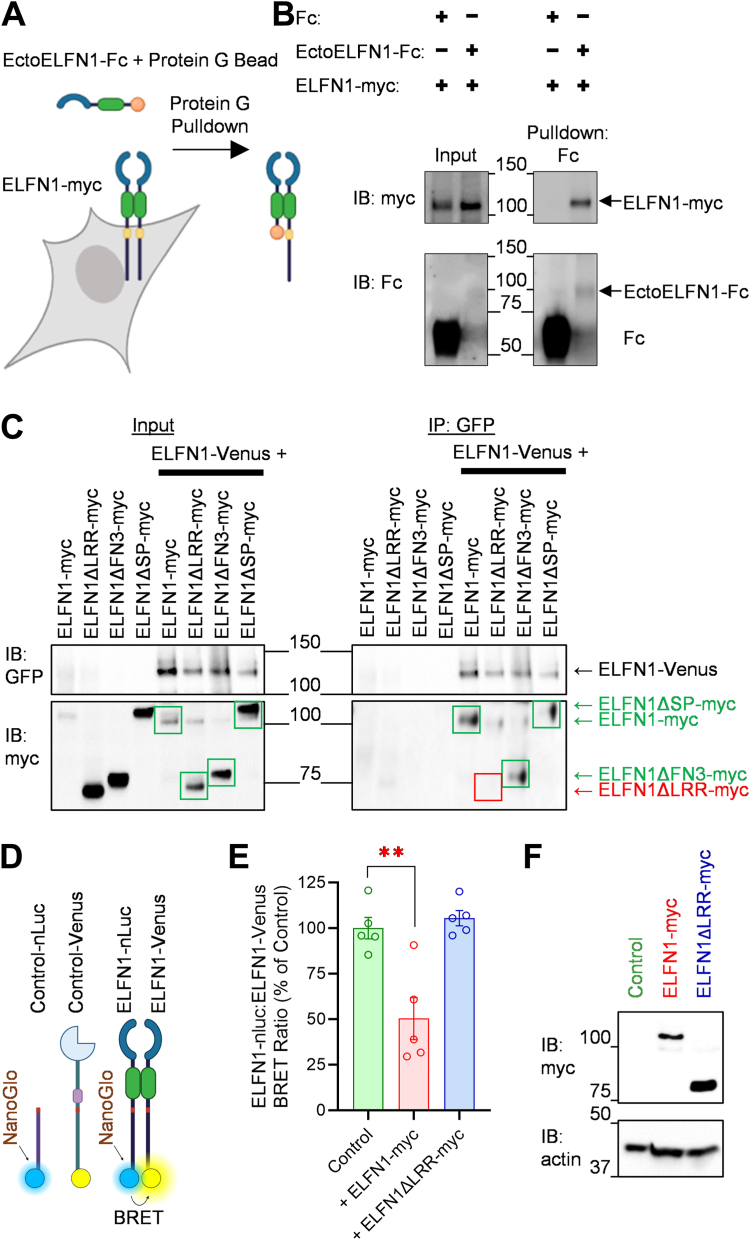
**ELFN1 homodimerization utilizes the extracellular LRR domain.***A,* Schematic representation of Protein G pulldown of ELFN1 extracellular domain (EctoELFN1-Fc) with full-length ELFN1-myc. *B,* Representative Western blots from 3 independent Protein G pulldowns with Fc or EctoELFN1-Fc secreted into media captured by protein G beads and mixed with HEK293 cell lysates expressing full-length ELFN1-myc. EctoELFN1-Fc is sufficient to pulldown ELFN1-myc: indicating the extracellular domain in homodimerization. *C,* Representative Western blots from 4 independent co-immunoprecipitation experiments using GFP antibody to immunoprecipitate ELFN1-Venus and evaluating co-immunoprecipitation of described myc-tagged ELFN1 constructs demonstrates lack of co-immunoprecipitation of ELFN1ΔLRR-myc suggesting LRR region requirement for dimerization. *D,* Schematic representation of BRET pairs used *(E)* while attempting the disrupt dimerization *via* competition by expressing either Control pcDNA3.1, ELFN1-myc, or ELFN1ΔLRR-myc. Notably, only ELFN1-myc could disrupt this BRET pair, suggesting disruption of dimerization and the requirement of ELFN1 LRR domains. *F,* Representative Western blot from 5 independent BRET experiments demonstrating comparable expression between ELFN1-myc and ELFN1ΔLRR-myc.

To further validate ELFN1 homodimerization, we performed a pulldown experiment with a secreted Fc-tagged ELFN1 ectodomain and analyzed its ability to pulldown the full-length ELFN1-myc (Fig. 4, *A* and *B*). In control experiments, Fc alone was unable to precipitate ELFN1-myc. However, EctoELFN1-Fc efficiently pulled down ELFN1-myc (Fig. 4*B*): indicating the sufficiency of the ELFN1 extracellular domain in ELFN1 homodimerization.

To narrow the extracellular domain requirements for ELFN1 homodimerization, we performed co-immunoprecipitation experiments between ELFN1-Venus and ELFN1-myc, or mutant constructs lacking the LRR domain (ELFN1ΔLRR-myc), lacking the FN3 domain (ELFN1ΔFN3-myc), or hosting an altered signal peptide and cleavage site (ELFN1ΔSP-myc) (6). Notably, ELFN1-myc, ELFN1ΔFN3-myc, and ELFN1ΔSP-myc were all co-immunoprecipitated by ELFN1-Venus (Fig. 4*C*); however, ELFN1ΔLRR-myc would not co-immunoprecipitate with ELFN1-Venus, suggesting the requirement of the LRR domain in ELFN1 homodimerization. To support the requirement of the LRR domain in ELFN1 dimerization and validate our previous BRET results (Fig. 3*C*), we co-expressed empty plasmid or ELFN1-myc in an attempt to disrupt the BRET ratios between ELFN1-nLuc and ELFN1-Venus (Fig. 4*D*) through competitive interaction. Indeed, co-expression of ELFN1-myc significantly lowered the BRET ratio between ELFN1-nLuc and ELFN1-Venus when compared to co-expression of empty plasmid control (Fig. 4*E*), suggesting ELFN1-myc was competing for dimerization with ELFN1-nLuc and ELFN1-Venus and disrupting the BRET pairs. Importantly, co-expression of ELFN1ΔLRR-myc at comparable levels to ELFN1-myc (Fig. 4*F*) did not disrupt ELFN1-nLuc:ELFN1-Venus BRET ratios (Fig. 4*E*), reinforcing that ELFN1 dimerization requires the LRR domain.

**Figure 5:**
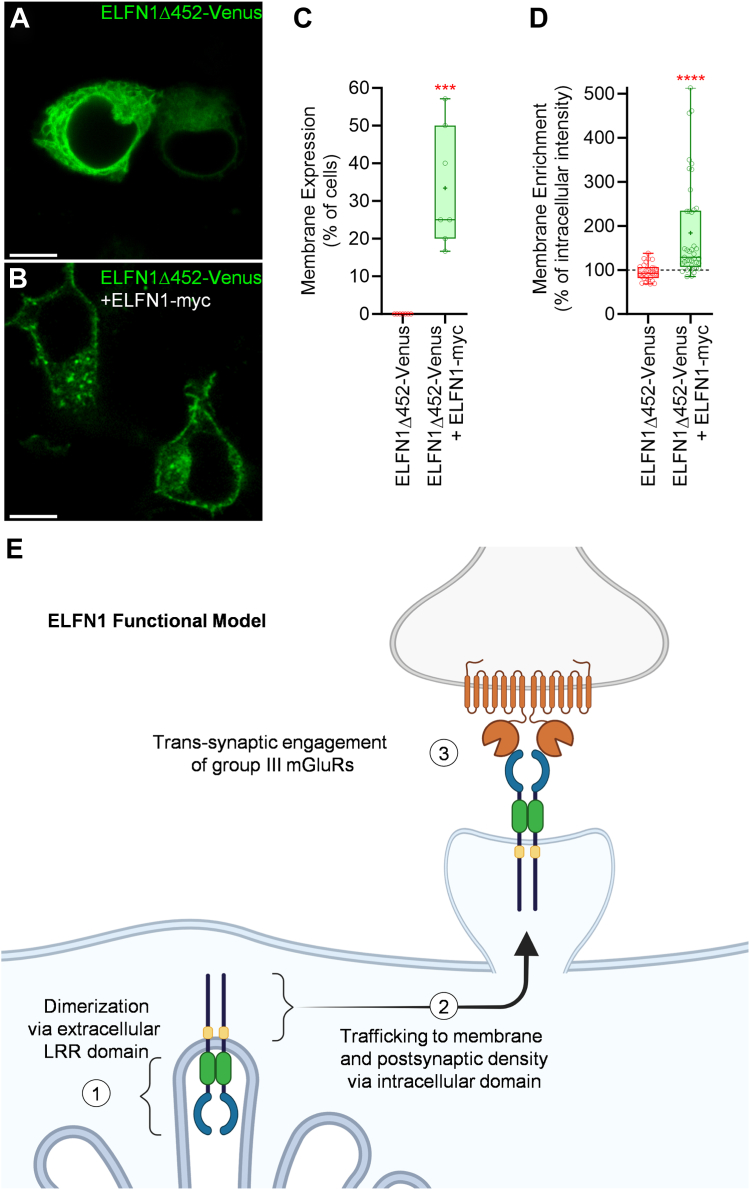
**ELFN1 dimerization *via* LRR domain precedes membrane expression and subsequent trans-synaptic engagement with group III mGluRs.***A,* ELFN1Δ452-Venus expressed in live HEK293 cells visualized using fluorescent confocal microscopy in the absence or *(B)* presence of ELFN1-myc (no fluorescence). *C,* Image quantification of percentage of cells exhibiting membrane enrichment for ELFN1Δ452-Venus (mean 0.000 ± SEM 0.000, n = 5 representing images from independent transfections) and ELFN1Δ452-Venus + ELFN1-myc (mean 33.40 ± SEM 5.943, n = 7), which were significantly different using a two-tailed, unpaired Mann-Whitney test (∗∗∗*p* value = 0.0001). *D,* Quantification of cell membrane enrichment of ELFN1Δ452-Venus under control conditions (median = 92.50%, n = 28 representing cells across 5 independent experiments) compared to co-expression of ELFN1-myc (median = 129.0%, n = 41 representing cells across 7 independent experiments) demonstrates significantly higher membrane enrichment of ELFN1Δ452-Venus in the presence of ELFN1-myc compared to control (∗∗∗∗*p* value = <0.0001) using a Mann-Whitney test due to failed normality test. These data collectively indicate that ELFN1 forms obligate dimers *via* the LRR domains prior to trafficking to the membrane, and only one ELFN1 juxtamembrane region is required for the dimer’s membrane trafficking. *E,* Proposed ELFN1 functional model of autoregulation, whereby (1) ELFN1 utilizes its extracellular LRR domains to form obligate homodimers prior to (2) trafficking to the membrane and post-synaptic density *via* the membrane-targeting domain (*yellow*) where (3) ELFN1 homodimers (or ELFN1/2 heterodimers) ultimately trans-synaptically engage pre-synaptic group III mGluRs to modulate synaptic properties, as previously described (6, 8, 10, 12, 13, 14).

We next examined the relationship between ELFN1 dimerization and its membrane targeting. To do this, we expressed ELFN1Δ452-Venus that does not traffic to the membrane (Fig. 2, *C* and *D*) but is capable of dimerizing with full-length ELFN1-myc (Fig. 3*D*) and evaluated the impact of ELFN1-myc co-expression on the localization of ELFN1Δ452-Venus using live-cell confocal fluorescent microscopy (Fig. 5*C*). Strikingly, while ELFN1Δ452-Venus expressed alone was never observed at the cell surface (Fig. 2, *C* and *D*; Fig. 5, *A* and *C*), its co-expression with full-length ELFN1-myc greatly facilitated its membrane localization (Fig. 5, *B* and *C*). Because membrane localization was rescued in a fraction of the cell population (Fig. 5*C*), we performed additional analyses to quantify the extent of membrane enrichment by comparing pixel intensity between the membrane and proximal cytosolic compartment and found a significant membrane enrichment of ELFN1Δ452-Venus when co-expressed with ELFN1-myc (Fig. 5*D*). Collectively, these observations suggest that formation of ELFN1 homodimers requires LRR domains and occurs prior to targeting dimeric ELFN1 protein to the plasma membrane and synaptic destinations (Fig. 5*E*).

### ELFN2 employs similar autoregulatory principles as ELFN1 and both proteins form heterodimers

**Figure 6:**
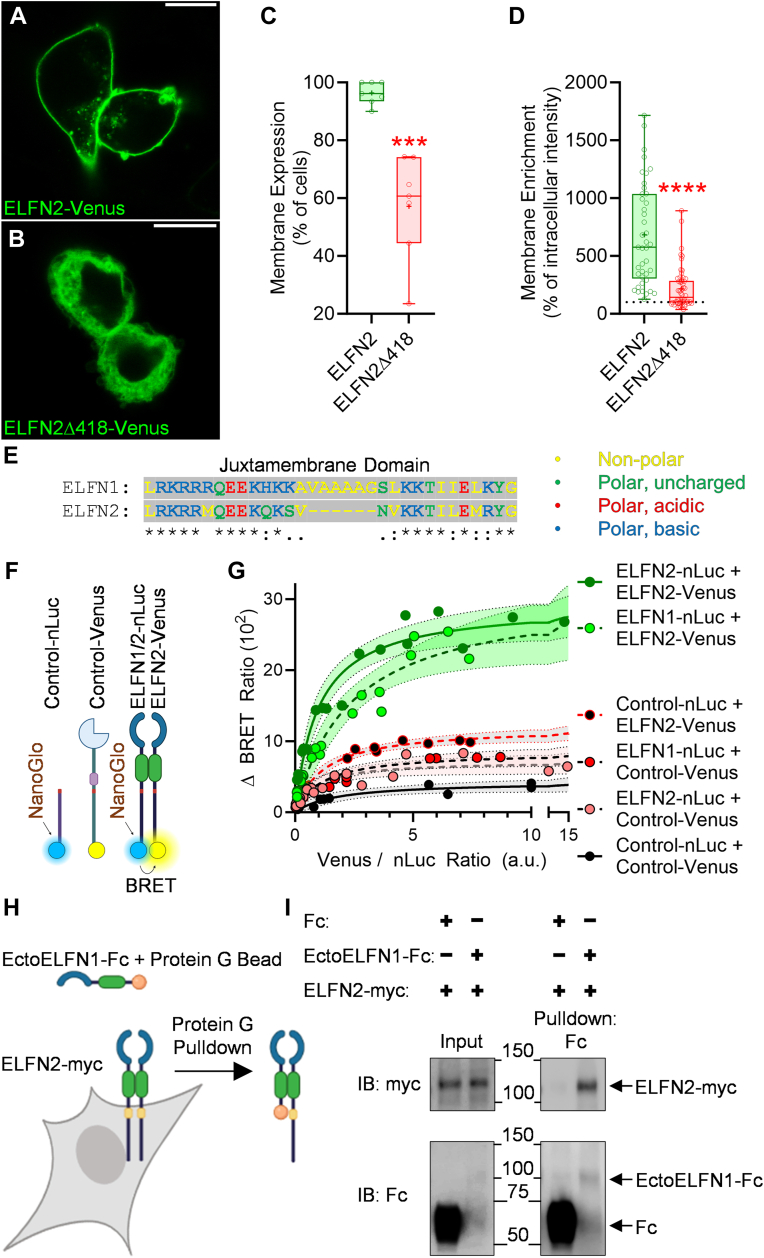
**ELFN2 employs similar autoregulatory principles as ELFN1 and both proteins form heterodimers.***A,* Representative images of ELFN2-Venus or *(B)* ELFN2-Δ418-Venus expressed in live HEK293 cells visualized using fluorescent confocal microscopy. *C,* Image quantification of percentage of cells exhibiting membrane enrichment. ELFN2-Venus (mean 96.38 ± SEM 1.467, n = 7 representing images from independent experiments) was significantly different than ELFN2Δ418-Venus (mean 57.17 ± SEM 6.809, n = 7) using a two-tailed, unpaired *t* test (∗∗∗*p* value < 0.001). *D,* Quantification of cell membrane enrichment of ELFN2-Venus (median = 575.0, n = 41 representing cells across 4 independent experiments analyzed) or ELFN2Δ418-Venus (median = 142.0, n = 56) demonstrated significantly lower membrane enrichment of ELFN2Δ418-Venus (∗∗∗∗*p* Value = <0.0001) using a Mann-Whitney test due to failed normality test, indicative of intracellular domain regulation of ELFN2 membrane expression. *E,* Sequence alignment of ELFN1 and ELFN2 intracellular juxtamembrane domains showing high homology. *F,* Schematic representation of nLuc- and Venus-tagged constructs used for *(G)* titration BRET experiments demonstrating increasing Control-Venus levels does not lead to an appreciable increase in BRET ratio with Control-nLuc (Bmax = 3.076–6.848). Neither control-nLuc with increasing ELFN2-Venus levels (Bmax = 10.79–13.57), ELFN2-nLuc with increasing Control-Venus levels (Bmax = 5.596–9.436), nor ELFN1-nLuc with increasing Control-Venus levels (Bmax = 7.202–10.23) were distinguishable from Control-nLuc with Control-Venus. ELFN2-nLuc transfected with increasing amounts of ELFN2-Venus exhibited a change in BRET ratio (Bmax = 25.75–33.68) distinguishably greater than Control-nLuc with Control-Venus suggestive of ELFN2 homodimerization, and ELFN1-nLuc transfected with increasing amounts of ELFN2-Venus exhibited a change in BRET ratio (Bmax = 25.20–40.61) distinguishable from Controls suggestive of ELFN1/ELFN2 heterodimerization. Each non-linear regression includes 8 to 16 independent values and error bars representing 95% confidence intervals. *H,* Schematic representation of Protein G pulldown of ELFN1 extracellular domain (EctoELFN1-Fc) with full-length ELFN2-myc. *I,* Representative Western blots from 3 independent experiments of Protein G pulldown with Fc or EctoELFN1-Fc secreted into media captured by protein G beads and mixed with HEK293 cell lysates expressing full-length ELFN2-myc.

To determine whether the principles that we describe for ELFN1 also apply to its closest homolog ELFN2, we engineered Venus-tagged ELFN2 constructs with intact or completely truncated carboxyl-termini: ELFN2-Venus and ELFN2Δ418-Venus, respectively. Using live-cell imaging on fluorescent confocal microscopy, ELFN2-Venus was found enriched at the membrane (Fig. 6, *A* and *C*) and ELFN2Δ418-Venus, which lacks the entire carboxyl terminus, exhibited a significant deficit in membrane localization (Fig. 6, *B* and *C*). Because a fraction of cells expressing ELFN2Δ418-Venus exhibited ELFN2Δ418-Venus membrane expression, we performed additional analyses for membrane enrichment using pixel intensities and found significantly lower levels of membrane enrichment for ELFN2Δ418-Venus when compared to ELFN2-Venus (Fig. 6*D*). Notably, ELFN1 and ELFN2 demonstrate high sequence homology in the intracellular juxtamembrane domains (Fig. 6*E*).

Next, we performed BRET-based ELFN2 homodimerization experiments (Fig. 6*F*) and found ELFN2-nLuc co-transfected with ELFN2-Venus exhibited a sharply increasing and saturating pattern in ΔBRET ratio suggestive of homodimerization: with higher BRET ratios to comparable controls (Fig. 6*G*). The substantial sequence homology between ELFN1 and ELFN2 (8) prompted us to test whether ELFNs can heteromerize. Therefore, we performed BRET-based ELFN1/ELFN2 heterodimerization experiments in parallel (Fig. 6*G*) and found comparably high and saturating ΔBRET ratios between ELFN1-nLuc and ELFN2-Venus with distinguishable amplitude from Control-nLuc and Control-Venus conditions (Fig. 6*G*). To validate this finding, we performed pulldown experiments utilizing the secreted ELFN1 ectodomain conjugated to Fc and found that ELFN2-myc was successfully captured by EctoELFN1-Fc but not Fc alone (Fig. 6, *H* and *I*): suggesting ELFN1 and ELFN2 share similar autoregulatory principles and form heterodimers *via* their extracellular domains.

## Discussion

Prior to this study, the understanding of ELFN1 molecular function was restricted to examining its ability to form trans-synaptic interactions with group III mGluRs, stabilize their expression at the synapse, and modulate their pharmacological activity (6, 8, 10, 12, 13): functions mediated by the extracellular domain of the protein. Although this remains the critical function of ELFN1 as a synaptic modulator, the role of the ELFN1 intracellular domain and its autoregulatory properties were completely unknown. We hypothesized that ELFN1 intracellular domain hosted autoregulatory functions to facilitate its ultimate engagement with group III mGluRs across the synapse. Indeed, truncation of the ELFN1 carboxyl domain inhibited ELFN1 membrane expression and postsynaptic localization: a critical first-step prior to trans-synaptic recruitment of group III mGluRs at the presynaptic terminal. Serendipitously, we discovered that an intracellular juxtamembrane region of ELFN1 plays a critical role in its localization, and that this region hosts numerous positively-charged amino acids consistent with the “positive inside” rule (22). Interestingly, this region showed homology to corresponding sequences present in receptor tyrosine kinases where it plays a role in their dimerization (20): prompting us to examine the dimerization status of ELFN1. Indeed, we observed that ELFN1 forms homodimers using multiple complementary techniques. Surprisingly, however, the juxtamembrane domain was not involved in this process and dimerization of ELFN1 was instead mediated by its extracellular domain: specifically, the LRR domain. Nevertheless, we found an intriguing connection between ELFN1 dimerization and trafficking as our experiments indicate that dimerization of ELFN1 precedes its surface targeting: as dimerization of intracellularly-trapped ELFN1 mutant with full-length ELFN1 is capable of rescuing plasma membrane localization of the dimeric complex. Thus, one intact membrane-targeting region within an ELFN protomer is sufficient for membrane expression of the homodimer. Whether this region has critical membrane associations or alternative roles will be the focus of future structural studies.

This study represents the first evidence that ELFN1 can homodimerize. Given that ELFN1’s trans-synaptic partners, group III mGluRs, are known to exist as obligate dimers (23, 24), our findings suggest that ELFN1-mGluR complexes at synapses may be organized at 2:2 stoichiometric arrangement of ELFN1 and mGluR protomers, though advanced structural studies would be required to address this hypothesis. Although preliminary binding-determinants between ELFN1 and group III mGluRs have been reported (6, 25), high-resolution structural studies would be required to conclusively address the formation and stoichiometry of these increasingly important trans-synaptic complexes. Nevertheless, ELFN1 utilizes the distal amino terminus and FN3 domain to interact with group III mGluRs (6), and we’ve now determined it utilizes the distinct LRR domains for self-association. Notably, LRR domains have been consistently implicated as dimerization interfaces for numerous cell adhesion molecules (26, 27, 28), including synaptic adhesion-like molecule 3 (SALM3) that similarly hosts an FN3 domain. Given the different domain requirements for ELFN1 dimerization and mGluR interactions, and that ELFN1 homodimerization experiments were done in the absence of group III mGluRs, it appears that ELFN1 homodimerization occurs independently of group III mGluR homodimerization. Whether and how ELFN1 dimers interact with constitutive mGluR homo and heteromers will be an interesting area of future investigation.

This study also represents the first documented function for the ELFN1 intracellular tail as both an essential domain for membrane trafficking and postsynaptic localization of ELFN1. Notably, multiple missense *ELFN1* mutations within the intracellular tail have been identified in a population of patients with epilepsy, attention-deficit/hyperactivity disorder, and/or autism spectrum disorders (12). Of note, some of these mutations (ELFN1-R650 C and ELFN1-D678 N) exhibited a modest ∼20% reduction in relative ELFN1 expression in hippocampal neuronal cultures (12): in alignment with the proposed autoregulatory role of the ELFN1 carboxyl tail. In fully removing this region in our study *via* truncation (ELFN1Δ600), we found ELFN1 is still capable of effectively trafficking to the membrane. This provokes the hypothesis that these intracellular disease-associated ELFN1 residues (amino acids ∼650–700) may contribute to alternative intracellular ELFN1 functions, perhaps related to autoregulation of expression as previously suggested (12); whereas the juxtamembrane intracellular ELFN1 region (amino acids 440–471) appears integral for ELFN1 trafficking.

Interestingly, we also find that ELFN2 exhibits similar autoregulatory properties to its homologous protein ELFN1. We demonstrate that truncation of the ELFN2 intracellular tail significantly impairs ELFN2 trafficking to the membrane; however, it was not completely lost as in the case of ELFN1. This suggests that ELFN2 may utilize additional mechanisms to facilitate membrane expression beyond the intracellular domain and suggests a minor functional distinction between ELFN1 and ELFN2. Indeed, sequence alignment of ELFN1 and ELFN2 demonstrate greater differences in the intracellular region, including in the proposed membrane-targeting motif (8). Nevertheless, we demonstrate that ELFN2 similarly forms homodimers, and together with ELFN1 form heterodimers *via* the extracellular domain: a concept reminiscent of roundabout (Robo) receptors that homo- and heterodimerize *via* their large extracellular domains (29, 30, 31). Given ELFN1 and ELFN2 appear to provide a similar functional regulation of group III mGluRs *in trans* (6, 8, 14), it is unlikely ELFN1/2 heterodimerization would alter group III mGluR function distinctly from ELFN1/2 homodimers. It is therefore tempting to hypothesize that the functional discrepancies of ELFN1/2 homodimers and heterodimers may lie subtly within the autoregulation of dimer trafficking dynamics.

On the basis of our findings, we propose the following model for ELFN1 function (Fig. 5*E*). According to this model, ELFNs form obligate dimers during protein synthesis *via* their amino termini. Following dimerization, ELFN dimers are trafficked to the membrane and postsynaptic density *via* their carboxyl termini: particularly the ∼30 amino acid juxtamembrane region that is integral for plasma membrane-targeting. After ELFN dimerization and postsynaptic-targeting, appropriately-localized postsynaptic ELFN dimers can trans-synaptically engage and stabilize presynaptic group III mGluRs to ultimately modulate synaptic properties: in agreement with previous studies on ELFN1 and ELFN2 (6, 8, 10, 11, 12, 13, 14).

In summary, we report novel autoregulatory functions of ELFNs. Given the recent increase in reports of pathogenic ELFN1 mutations (15, 16), these studies provide new readouts to evaluate ELFN1 functionality and identify regions of interest for prospective ELFN1 mutations for predicting deleterious functions. Future studies will be focused on further characterizing the elusive postsynaptic functions of ELFN1 beyond the autoregulatory roles described herein.

## Experimental procedures

### Animals

Studies involving animals were approved by the IACUC committee at the Herbert Wertheim UF Scripps Institute for Biomedical Innovation & Technology. Wild type C57Bl6 mice were purchased from Charles River and were used as source of brain tissues.

#### cDNA plasmids

ELFN1-myc was previously described (13). ELFN1Δ734-myc, ELFN1Δ600-myc, ELFN1Δ536-myc, ELFN1Δ501-myc, ELFN1Δ471-myc, ELFN1Δ452-myc, and ELFN1Δ439-myc are truncations of the ELFN1-myc construct generated *via* In-Fusion cloning (Takara Bio). ELFN1-nLuc, ELFN1-Venus, ELFN1Δ734-Venus, ELFN1Δ600-Venus, ELFN1Δ536-Venus, ELFN1Δ501-Venus, ELFN1Δ471-Venus, ELFN1Δ452-Venus, ELFN1Δ439-Venus, ELFN2-nluc, ELFN2-Venus, and ELFN2Δ418-Venus were generated using In-Fusion cloning (Takara Bio) by inserting an nLuc or Venus sequence between the myc sequence and the stop codon. ER marker mCherry-SEC61 B was obtained from Addgene (Plasmid #121160). ELFN1ΔLRR-myc, ELFN1ΔFN3-myc, and ELFN1ΔSP-myc were described previously (6, 32). Control-Venus (GPR158-Venus) and Control-nLuc (masGRK3ct-nLuc), described previously (33), were used due to their membrane-localization and comparably sized carboxyl termini to ELFN1. Fc and EctoELFN1-Fc constructs were described previously (13). ELFN2-myc was previously described (8).

#### Cell culture

HEK 293 T/17 cells were cultured in Dulbecco’s modified Eagle’s medium supplemented with 10% fetal bovine serum, minimum essential medium nonessential amino acids (Life Technologies), 1 mM sodium pyruvate, penicillin (100 U/ml), and streptomycin (100 μg/ml) at 37 °C in a humidified incubator containing 5% CO_2_. For each HEK 293 T/17 cell experiment, cells were seeded without penicillin and streptomycin and transfected the following day at ∼70% confluency. Cells were transiently transfected with the appropriate expression constructs, as labelled in figures, using Lipofectamine LTX with Plus Reagent. The empty vector pcDNA3.1 was used to normalize the amount of DNA in each transfection. Primary mouse cortical neurons were harvested from P0 C57BL/6 pups by isolating the cortices, washing in cold HBSS, and digesting for 15 min at 37 C in a digestion buffer (137 mM NaCl, 5 mM KCl, 7 mM Na_2_HPO_4_, 25 mM HEPES, pH 7.2) supplemented with 0.3 mg/ml Papain (Worthington) immediately before use. Dissociated cells were then washed in 20% fetal bovine serum (FBS) in HBSS 3 times, followed by HBSS 3 times, followed by growth media 3 times (Neurobasal A supplemented with GlutaMAX, B27, and PenStrep (Life Technologies)). DNAse I (Life Technologies) was supplemented in the final wash and cells were triturated and counted for plating into Poly-D-Lysine-coated glass-bottomed dishes. Cells were incubated at 37 C at 5% CO_2_ and half media change was performed without antibiotics after 2 days, and every 3 days thereafter. Cells were transfected with ELFN1-Venus or ELFN1Δ439-Venus using Lipofectamine 2000.

#### Confocal fluorescent microscopy

Microscopy experiments were performed on a Nikon A1R Confocal Microscope on a Ti-E Inverted Microscope or an Olympus FV3000 Confocal Laser Scanning Microscope for Figure 2*B*. HEK 293 T/17 cell experiments were performed in PDL-coated glass-bottomed dishes on live cells using the 514 (Nikon) or 488 (Olympus) laser to excite Venus and 561 laser to excite mCherry. Representative images of multiple cells across multiple experiments were taken and quantification was performed as the percentage of cells per trasfection that exhibited membrane enrichment, with 5 to 12 images per condition, or colocalization with mCherry-SEC61 B, with 4 to 9 images per condition. Marked qualitative changes in membrane enrichment were assessed visually; however, further analyses on membrane enrichment was provided by comparing pixel intensity at the membrane to intracellular pixel intensity proximal to the membrane using ImageJ. Primary cortical neurons were fixed and permeabilized with 4% paraformaldehyde and 0.1% Triton-X following transfection with ELFN1-Venus or ELFN1Δ439-Venus and labelled for endogenous PSD-95 (Cell Signaling, 3450) with Alexa Fluor 647 (Invitrogen A-21245) for wide separation. Images were taken from multiple neuronal preparations focused on dendritic projections identified with endogenous PSD-95 puncta. Each projection was subsequently isolated into separate images and quantified using the percentage of endogenous PSD-95 colocalizing with ELFN1-Venus or ELFN1Δ439-Venus, with 19 images per condition. All colocalization data were calculated using JACoP Plugin for ImageJ.

#### Multiple sequence alignment

Multiple sequence alignment was performed using Clustal Omega on 26 mammalian ELFN1 sequences. Sequences included had similar length to mouse ELFN1 and had an annotation score of 2 or higher on UniProt. Sequences with unique major deletions were excluded from the multiple sequence alignment. Species included were: *Mus musculus* (Mouse), *Homo sapiens* (Human), *Pan troglodytes* (Chimpanzee), *Gorilla gorilla* gorilla (Western lowland gorilla), *Pongo abelii* (Sumatran orangutan), *Rhinopithecus roxellana* (Golden snub-nosed monkey), *Chlorocebus sabaeus* (Green monkey), *Mandrillus leucophaeus* (Drill), *Cercocebus atys* (Sooty mangabey), *Papio anubis* (Olive baboon), *Macaca nemestrina* (Pig-tailed macaque), *Bos taurus* (Bovine), *Sus scrofa* (Pig), *Delphinapterus leucas* (Beluga whale), *Cavia porcellus* (Guinea pig), *Felis catus* (Cat), *Canis lupus* familiaris (Dog), *Aotus nancymaae* (Ma's night monkey), *Monodelphis domestica* (Gray short-tailed opossum), *Ursus americanus* (American black bear), *Vulpes vulpes* (Red fox), *Mustela putorius* furo (European domestic ferret), *Vombatus ursinus* (Common wombat), *Sarcophilus harrisii* (Tasmanian devil), *Mesocricetus auratus* (Golden hamster), and *Cricetulus griseus* (Chinese hamster). Conserved residues are represented in grey.

#### AlphaFold model

Mouse ELFN1 sequence was used (Structure AF-Q8C8T7-F1). Sequence from amino acid 440 to 471 was highlighted. Residues within a helical model were depicted in blue, and residues disordered were highlighted in red. Model confidence values were reported as a per-residue confidence score, or the predicted Local Distance Difference Test (pLDDT) with >90 being very high confidence, 70 to 90 being confident, 50 to 70 being low confidence, and <50 being very low confidence.

#### MotifFinder

Mouse ELFN1 sequence was input into MotifFinder (www.genome.jp/tools/motif) and conserved domains overlapping with the 440 to 471 region were sought using the NCBI conserved domain database. An E-value cut-off score of 1.0 was used. After identifying TM-ErbB2 with significant overlap, this alignment with human ErbB2 was extended to include more ELFN1 membrane-targeting domain for comparison.

#### BRET dimerization experiments

Transfections were performed using constructs described, using stable amounts of nLuc expression constructs (5 ng) and increasing amounts of Venus expression constructs (0–2.4 μg). Promega Nano-Glo Luciferase Assay Reagent was added to the cells to 0.1% and BRET ratios were calculated as the ratio of Venus emissions (535 nm) to nLuc emissions (460 nm) captured on a BMG LabTech PHERAstar FSX plate-reader. Raw Venus readings were also captured separately by exciting Venus with a 488 nm laser in the absence of Nano-Glo reagent to determine total Venus expression. Data was expressed with the raw Venus value/nLuc in arbitrary units (a.u.) on the x-axis, and change in BRET ratio (Δ BRET ratio) on the y-axis. Data represents 4 independent experiments run in parallel, and are therefore directly comparable across graphs. Data is presented with 95% confidence intervals for error bars and Bmax representing max amplitudes. BRET disruption experiments were performed on a BioTEK Synergy Neo2 plate-reader with stable levels of ELFN1-nLuc and ELFN1-Venus below saturation: varying only co-expression of pcDNA3.1, ELFN1-myc, or ELFN1ΔLRR-myc that possess neither donor or acceptor molecules. Comparable expression of ELFN1-myc and ELFN1ΔLRR-myc was confirmed using Western blotting.

#### Co-immunoprecipitations

Co-transfections in HEK 293 T/17 cells were performed with the described constructs (2.5 μg of each). Cells were lysed with a 1% Triton-X lysis buffer and insoluble material was pelleted. Lysates were incubated with Protein G Sepharose Beads (Cytiva) conjugated with mouse GFP antibody (MA5-15256) for 1 h rotating at 4 C and washed with lysis buffer 3 times *via* centrifugation. Lysates and beads were treated with β-mercaptoethanol-containing sample buffer and SDS-PAGE and Western blotting was performed using 10% milk blocking, then mouse GFP antibody or rabbit myc antibody (Genscript A00172) in 3% milk at 1:1000. Secondary antibodies were purchased from Jackson ImmunoResearch and used at 1:10,000 in 3% milk. Images were obtained using a KwikQuant Imager and KwikQuant ECL Solutions (Kindle Biosciences), or ChemiDoc Imaging System and Clarity and Clarity Max ECL Western Blotting Substrates (Bio-Rad).

#### Protein G pulldowns

Transfections in HEK 293 T/17 cells were performed individually in 3 separate cell populations. Fc and EctoELFN1-Fc conditions had their media replaced with OPTI-MEM to allow for the accumulation of secreted proteins. After 48 h, OPTI-MEM was collected and incubated with Protein G Sepharose Beads (Cytiva) and cell lysates from ELFN1-myc or ELFN2-myc transfected cells in 1% Triton-X Lysis Buffer rotating for 1 h at 4 C. Samples were washed three times with lysis buffer *via* centrifugation before given sample buffer containing β-mercaptoethanol. Inputs were prepared separately and SDS-PAGE and Western blotting was performed using 10% milk blocking, then mouse Fc antibody (ThermoFisher Scientific EM-07) or rabbit myc antibody (Genscript A00172) in 3% milk at 1:1000. Secondary antibodies were purchased from Jackson ImmunoResearch and used at 1:10,000 in 3% milk. Images were obtained using a KwikQuant Imager and KwikQuant ECL Solutions (Kindle Biosciences).

#### Statistics

Normality was tested using the Kolmogorov-Smirnov test to guide appropriateness of statistical tests. Comparison of two datasets passing the normality test utilized a two-tailed, unpaired *t* test. Comparison of two datasets failing the normality test utilized a two-tailed, unpaired Mann-Whitney test. Comparison of more than two datasets utilized one-way ANOVA with multiple comparisons test. Multiple comparisons failing the normality test utilized the Kruskal-Wallis test and Dunn’s multiple comparisons test.

#### Schematic preparations

Created with Biorender.com.

## Data availability

All data are contained within the manuscript. Further data sharing is available upon request *via* corresponding authors (e-mails provided).

## Conflict of interest

The authors declare that they have no conflicts of interest with the contents of this article.
